# The Functions of Myosin II and Myosin V Homologs in Tip Growth and Septation in *Aspergillus nidulans*

**DOI:** 10.1371/journal.pone.0031218

**Published:** 2012-02-16

**Authors:** Naimeh Taheri-Talesh, Yi Xiong, Berl R. Oakley

**Affiliations:** 1 Department of Molecular Biosciences, University of Kansas, Lawrence, Kansas, United States of America; 2 Department of Molecular Genetics, The Ohio State University, Columbus, Ohio, United States of America; University of Wisconsin - Madison, United States of America

## Abstract

Because of the industrial and medical importance of members of the fungal genus *Aspergillus*, there is considerable interest in the functions of cytoskeletal components in growth and secretion in these organisms. We have analyzed the genome of *Aspergillus nidulans* and found that there are two previously unstudied myosin genes, a myosin II homolog, *myoB* (product = MyoB) and a myosin V homolog, *myoE* (product = MyoE). Deletions of either cause significant growth defects. MyoB localizes in strings that coalesce into contractile rings at forming septa. It is critical for septation and normal deposition of chitin but not for hyphal extension. MyoE localizes to the Spitzenkörper and to moving puncta in the cytoplasm. Time-lapse imaging of SynA, a v-SNARE, reveals that in *myoE* deletion strains vesicles no longer localize to the Spitzenkörper. Tip morphology is slightly abnormal and branching occurs more frequently than in controls. Tip extension is slower than in controls, but because hyphal diameter is greater, growth (increase in volume/time) is only slightly reduced. Concentration of vesicles into the Spitzenkörper before incorporation into the plasma membrane is, thus, not required for hyphal growth but facilitates faster tip extension and a more normal hyphal shape.

## Introduction

Filamentous fungi in general, and members of the genus *Aspergillus* in particular, are important industrially and medically. They are important for the production of products as diverse as soy sauce, glucoamylase, a key enzyme in producing high fructose corn syrup, and anti-cholesterol drugs such as lovastatin. They also produce toxins such as aflatoxins, and some species are responsible for huge numbers of deaths of immune-compromised patients. There is, thus, considerable interest in understanding their biology in general, and their mechanisms of growth and secretion in particular.

The actin cytoskeleton is essential for several critical functions in filamentous fungi including tip growth, septation, endocytosis and exocytosis [1]–[10]. Many of the functions of the actin cytoskeleton are carried out through the interaction of actin microfilaments with motor molecules called myosins. Although many families of myosins exist (35 by one analysis [11]), the myosin families in fungi are generally limited. For example, the budding yeast *Saccharomyces cerevisiae* and the fission yeast *Schizosaccharomyces pombe* each have five myosin genes that fall into three myosin families [12]. Three genes encoding myosin heavy chain related proteins have been studied in *Aspergillus nidulans*. The *myoA* gene encodes a type I myosin that is essential for viability, polarized growth and secretion and also plays an important, perhaps essential, role in endocytosis [2], [4], [5]. In addition, there are two genes in which chitin synthase sequences are fused to myosin motor domains, *csmA* and *csmB* [13], [14]. The products of these genes belong to an unusual class of myosins, sometimes designated class 17 myosins [11] that is apparently restricted to fungi, Neither *csmA* nor *csmB* is essential, but null mutations of either gene cause morphological defects consistent with improper cell wall formation. In addition, deletion of *csmB* and simultaneous downregulation of *csmA* is lethal [14].

Many questions remain to be answered about myosin function and, in particular, about the roles that myosins play in tip growth and septation. In order for hyphae to grow, vesicles carrying wall precursors must move to the hyphal tip and undergo exocytosis, releasing their contents [6], [8]. Likewise endocytosis is probably also required for growth (discussed in [10]). Vesicles traffic through an organelle called the Spitzenkörper that is thought to act as a vesicle supply center [6], [8]. While MyoA appears to be the key myosin in endocytosis and exocytosis at the hyphal tip, the roles of myosins in vesicular trafficking along hyphae and in moving vesicles to the Spitzenkörper remain to be determined as do the identities of myosins involved in septation and their roles.

The sequencing of the *A. nidulans* genome presented the possibility of identifying and characterizing the functions of all remaining myosin heavy chains in *A. nidulans*. *A. nidulans* has two previously uncharacterized myosin heavy chain genes, one (here designated *myoB*) encoding a member of the myosin II family and the other (here designated *myoE*) encoding a myosin V family member. We have analyzed the functions of these genes and their products by deleting them and observing the phenotypes of the deletions and by tagging them with fluorescent protein sequences and observing them in living cells by time-lapse microscopy. We have found that neither is essential for viability, but each carries out important functions related to growth. In addition to completing the characterization of myosin heavy chains in *A. nidulans*, our results have important implications with respect to the mechanisms of tip growth and cytokinesis.

## Results

### Identification of *A. nidulans* myosin II and myosin V homologs

The existence of myosin II and myosin V genes in *A. nidulans* has been noted previously [11], [15], (note: in the Odronitz and Kollmar study [11] an alternative genus name, *Emericella*, was used and the genes were called mhc and myo5) but no analysis of the genes nor their functions was reported and the genome database numbers for the genes were not given. To identify these genes and determine if there were additional unidentified myosin heavy chain genes, we carried out BLAST (basic local alignment search tool) (http://www.ncbi.nlm.nih.gov/blast/Blast.cgi) searches of the *A. nidulans* genome databases (http://www.aspgd.org/, http://www.cadre-genomes.org.uk/, http://www.broadinstitute.org/annotation/genome/aspergillus_group/MultiHome.html) with multiple myosin sequences. We found the previously characterized myosin heavy chains and two previously uncharacterized myosin heavy chain genes, AN4706 and AN8862. AN4706 is predicted to encode a protein of 2404 amino acids. A BLAST search of the NCBI database with the predicted product of AN4706 revealed that almost all of the 100 proteins with the greatest similarity to the predicted product of AN4706 were type II myosin heavy chains or predicted type II myosin heavy chains and all had an E value of 0. Interestingly, the vertebrate myosins with the greatest identity to the predicted product of AN4706 were smooth muscle type II myosins. The predicted product of AN4706 aligned well with type II myosins over most or all of its length. An InterProScan (http://www.ebi.ac.uk/interpro/index.html) analysis indicates that it contains an N-terminal motor domain (myosin head), SH3-like domain, an IQ calmodulin-binding motif, a chromosome segregation ATPase region (SMC, structural maintenance of chromosomes) and a C-terminal myosin tail domain (Figure S1). These data and our results (below) leave no doubt that the product of AN4706 is a type II myosin heavy chain. Following the standard *A. nidulans* gene naming system, we hereby designate AN4706 as *myoB* and its product as MyoB.

AN8862 encodes a predicted product of 1569 amino acids. An InterProScan analysis reveals that it contains an N-terminal myosin head (motor domain), and a C-terminal tail region including a Dilute domain (Figure S1). A BLAST search of the NCBI database revealed that all top matches were type V myosin heavy chains. (Note that the nomenclature for type V myosins can be confusing. The *S. cerevisiae MYO2* gene, for example, encodes a type V myosin.) The top 100 matches had E values of 0 and showed strong identity with the predicted product of AN8862 over all or nearly all of its length. Based on these data and our results (below), we designate AN8862 *myoE* and its product MyoE. Figure S1 shows the predicted domain structure of all five *A. nidulans* myosin heavy chains.

### Deletions of *myoB* and *myoE* confer strong growth phenotypes

**Figure 1:**
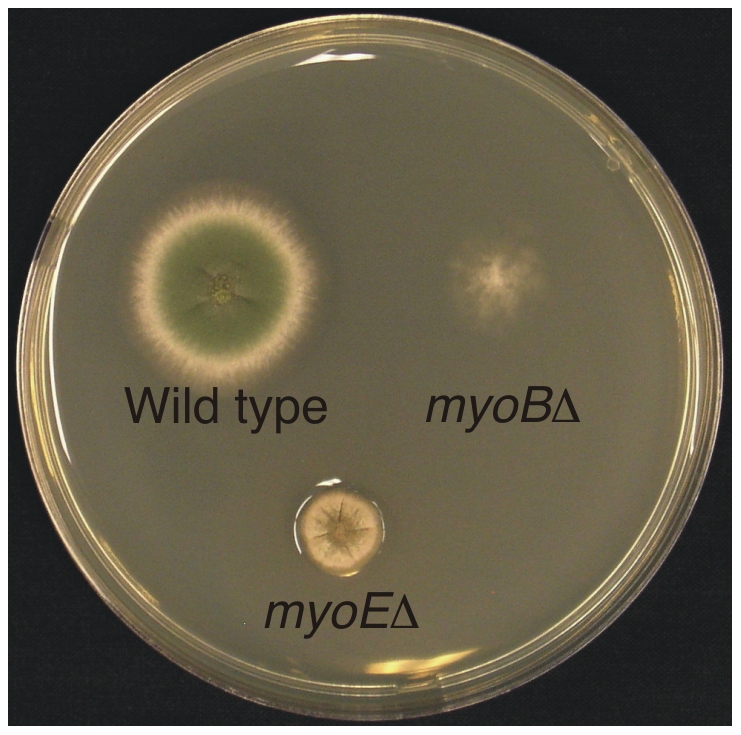
Growth phenotype of myosin deletants. Incubation was for three days at 37°C on YAG medium supplemented with riboflavin. While both *myoB* and *myoE* deletants are viable, the *myoB* deletant colony is thin and wispy. Microscopic examination revealed that individual hyphae extend beyond the apparent edge of the colony. The *myoE* deletant is compact, exhibiting slower radial growth than the control strain.

We deleted the *myoB* and *myoE* genes by replacing each of them with the *Aspergillus fumigatus pyrG* gene. (Genotypes of all strains are listed in Table S1.) Strains carrying deletions of each gene were viable, but both had strong growth phenotypes (Figure 1). The *myoB* deletion formed very thin, wispy, irregularly shaped colonies with a nearly complete absence of conidia. Because of the poor growth and conidiation we were not able to maintain permanent stocks of the *myoB* deletion. The *myoE* deletion (LO1833) produced compact colonies, the radial growth rate of which was less than half of that of the parental strain.

### MyoB forms nodes or strings that localize to the septum

To localize MyoB *in vivo*, we fused a GFP coding sequence [16] in frame to the 3′ end of the *myoB* coding sequence. We inserted the fragment into the genome at the *myoB* locus creating strain LO1973. The GFP-tagged *myoB* was the only copy of the *myoB* gene in the genome; it was under control of its endogenous promoter and it supported normal growth and conidiation over a wide range of temperatures (Figure S2).

**Figure 2:**
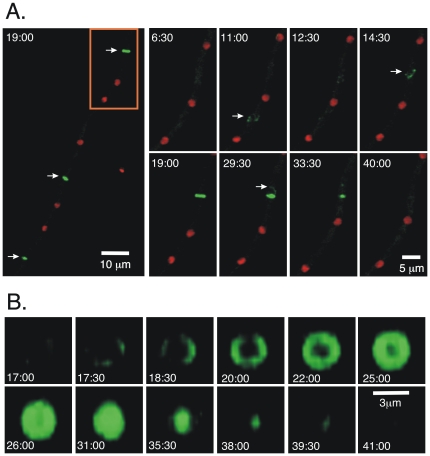
MyoB-GFP localization during septum formation. Images are projections from a time-lapse data set taken with strain LO2390. Times are in min and sec after the start of imaging. In A, the panel at the left is a low magnification image from the 19:00 min time point showing MyoB-GFP localization at three forming septa (arrows). Higher magnification time-lapse images of the region in the rectangle are shown to the right. The time after the beginning of time-lapse acquisition (in min) is shown at the upper left of each panel. In this set of images, strings of MyoB-GFP begin to coalesce at the 11 min time point (arrow) but then disperse and coalesce at a different place (arrow at 14:30). A septum then forms and begins to contract. Strings of MyoB-GFP can be seen leaving the septum (arrow at 29:30). Panel B is from the same time-lapse data set as A, but a single septum is shown and it is rotated 90° (using Volocity software) such that we have an end-on view of septum formation. MyoB-GFP assembles into a ring, with no evidence of it being spun out from a single spot. The ring then fills and contracts before disappearing. The time (in min) after the beginning of acquisition is shown at the bottom of each panel.

To visualize nuclei and MyoB-GFP in the same strain, we fused an mCherry sequence to the 3′end of the histone H1 gene in LO1973, creating strain LO2390. MyoB-GFP localized to forming septa in an interesting way (Figure 2). Septation in *A. nidulans* has been studied extensively, [17]–[20], and it is worthwhile to summarize it briefly. When conidia germinate, septation normally does not occur until after the third mitotic division (eight nuclei stage). Septation then occurs after each subsequent mitotic division. In mature, rapidly growing hyphae several septa often form after a round of mitosis and septation is asymmetrical, leaving a large multinucleate tip cell. The nuclei in the tip cell continue to go through the cell cycle, but nuclei in subapical cells are removed from the cell cycle until a side branch emerges.

After mitosis and immediately before septation, MyoB nodes or strings appeared between a subset of nuclei (Figure 2A, Video S1). They then coalesced to form a ring at the site of septation (Figure 2B, Video S2). The ring then constricted and MyoB moved away, again in strings (Figure 2A,B, Video S1, S2). Occasionally the strings would begin to coalesce between two nuclei and then move to a different position between two other nuclei before forming a ring (Figure 2A, Video S1). As will be discussed, this localization pattern has implications for the mechanism of septation. We noted that the process of septation generally occurred roughly equidistant from the nearest two nuclei and we saw no specific localization of MyoB at the hyphal tip. The MyoB localization at the forming septum occurred prior to the septum becoming visible by transmitted light.

### MyoB is critical for septum formation and correct chitin deposition

**Figure 3:**
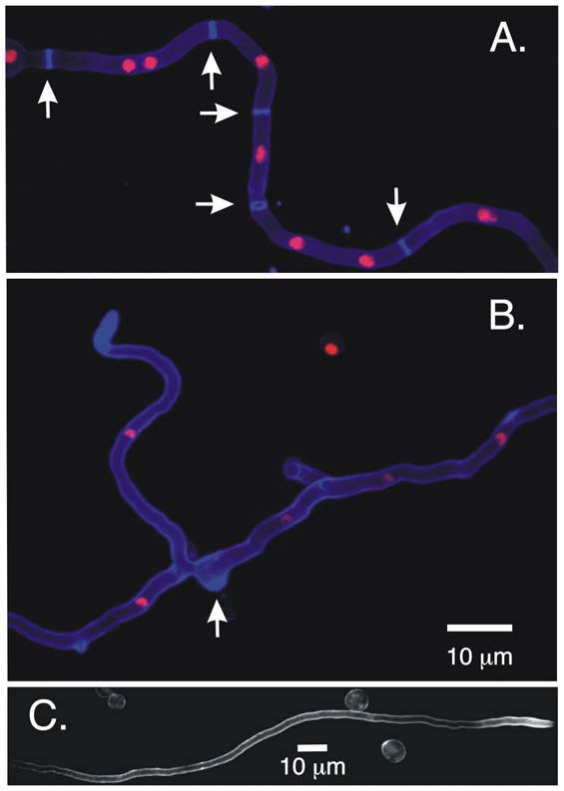
Deletion of myoB inhibits septum formation. All panels are images of living cells. In A and B, nuclei are shown with histone H1-mRFP and chitin is stained with calcofluor (10 µg/ml). A. a *myoB* ^+^ strain (LO1516). Multiple septa are visible (arrows). B. a *myoB*Δ hypha. The *myoB* gene was deleted in LO1516 and nuclei carrying the deletion were maintained in a heterokaryon. No septa are present but there are thickened regions containing chitin (e.g. arrow) and chitin is highly concentrated near the hyphal tip. C. Shows a hyhal tip region in a *myoB*Δ strain stained with calcofluor but nuclei are not imaged. Note the absence of septa and side branches. The circular objects are ungerminated conidia resulting from the heterokaryon rescue technique.

The *myoB* deletion produced almost no conidia and was sufficiently sick that it was difficult to work with. Fortunately in *A. nidulans* the heterokaryon rescue technique [21], [22]–[25] allows one to maintain nuclei carrying recessive sick or lethal mutations and to produce conidia carrying the mutations for microscopic analysis. Using this technique, we were able to analyze the phenotype of *myoB*Δ in detail. Since MyoB localizes specifically to septa, we examined the effects of *myoB*Δ on septation by germinating *myoB*Δ and control spores and staining them with calcofluor, which stains chitin-containing cell walls and septa [1]. We found that septation was almost completely abolished in *myoB*Δ strains (Figure 3). The rare septa that were seen were malformed. MyoB, thus, is critically important for septation. Our calcofluor staining also revealed that in the *myoB*Δ strain there were abnormal accumulations of chitin in hyphae (Figure 3). We also noted that virtually all *myoB*Δ hyphae had long stretches at the hyphal tip in which side branches were absent (Figure 3C), but since branching normally occurs in sub-apical cells, separated from the hyphal apex by one or more septa, we interpret the reduced branching to be a consequence of reduced septation.

### MyoB is not required for tip extension

The term “tip growth rate” is often used to denote the change in hyphal length per unit of time. We have found, however, that myosin deletions can affect both the rate of hyphal lengthening and the diameter of hyphae. We will consequently use the more precise terms “hyphal tip extension rate” for the change in hyphal length per unit of time and “hyphal growth rate” for the change in volume of the hyphal tip per unit of time. *myoB* deletants and parental control cells were grown in the same liquid medium at 25°C for 24–28 h. Z-series stacks were then captured at three-min intervals and tip extension rates were determined as described previously [26]. The tip extension rate in the *myoB*Δ strain (0.98±0.30 µm/min, n = 10), however, was significantly faster than in the control (0.65±0.18 µm/min, n = 10) (p = 0.0084, paired *t*-test). We also determined the mean hyphal diameter and found that the diameter for the *myoB*Δ strain was 2.56±0.19 µm (mean ± standard deviation, n = 10) vs 2.52±0.21 µm (n = 11) for the *myoB* ^+^ control. The diameters of *myoB*Δ and *myoB* ^+^ strains are, thus, essentially identical. MyoB, thus, is not required for tip extension, and, indeed, tip extension rates and hyphal growth rates are greater in its absence. As will be discussed, this may relate to the number of nuclei in the tip cell and the size of the cell.

### MyoE localizes to moving puncta in the cytoplasm and to the Spitzenkörper

**Figure 4:**
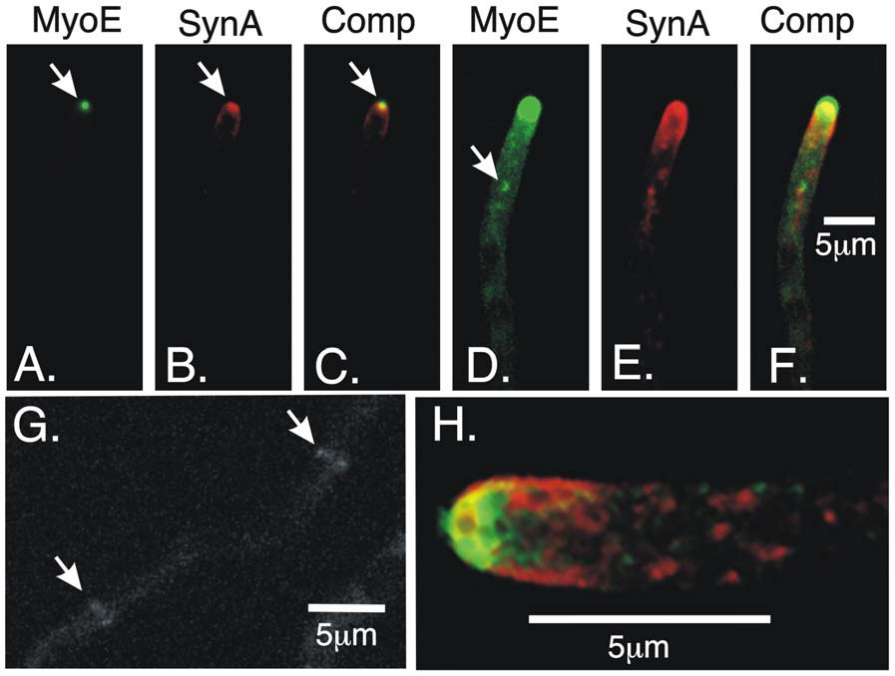
MyoE localization. A–F are images of the same field and are maximum intensity projections of a Z-series stack. A–C show the co-localization of MyoE-GFP and mCherry-SynA at the Spitzenkörper (arrows). SynA localizes to the Spitzenkörper and to the plasma membrane near the apex (B). In D–F, the thresholds are chosen to reveal the punctate staining in the hypha while overexposing the MyoE-GFP and mCherry-SynA at the hyphal tip. MyoE-GFP localizes to numerous small puncta and some larger structures that may be endosomes (e.g. arrow). G. Faint localization of MyoE-GFP at forming septa (arrows). H. A three-dimensional projection of a hyphal tip showing MyoE-GFP and mCherry-SynA. Although MyoE and SynA co-localize at the Spitzenkörper, many puncta behind the tip show GFP fluorescence or mCherry fluorescence, but it was not clear that there was any obligate co-localization.

To determine the localization patterns of MyoE *in vivo*, we fused the GFP coding sequence in frame to the 3′ end of *myoE* gene, creating strain LO1975. The *myoE*-GFP fusion was the only copy of the *myoE* gene in the genome, it was under control of the normal *myoE* promoter and it supported normal growth over a wide range of temperatures (Figure S3). MyoE was most concentrated at the hyphal apex at a position corresponding to the Spitzenkörper (Figure 4A). The Spitzenkörper (literal translation is “tip body”) is an incompletely characterized organelle at hyphal apices that plays a key role in tip growth [6], [8], [27]. Vesicles containing materials for tip growth traffic through it and it is thought to be a vesicle supply center for tip growth [28]. The Spitzenkörper can be visualized in *A. nidulans* by using fluorescently tagged SynA. SynA is a synaptobrevin homolog and is a marker for exocytic vesicles [10] and endosomes [29]. It concentrates in the hyphal tip area and is most concentrated at the Spitzenkörper [10]. It also localizes to the plasma membrane between the extreme hyphal apex and a ring of endocytic sites [10]. MyoE co-localized with SynA specifically at the Spitzenkörper but not at the plasma membrane (Figure 4 A–C).

MyoE-GFP also localized to many dots in the cytoplasm, some of which were at the limit of resolution and some of which were slightly larger (Figure 4D). Most of the dots were mobile (Video S3). Their density and rapidity of movement made it difficult to track individual MyoE dots but it was apparent that they moved bi-directionally, toward and away from the hyphal tip, they moved at different rates and they often paused. MyoE also localized very weakly and transiently to forming septa (Figure 4G).

MyoE is expected to be involved in the movement of vesicles to the hyphal tip [8] and the punctate distribution of MyoE-GFP suggested that it might localize to vesicles. In an attempt to determine if this was the case, we performed rapid dual wavelength imaging of strain LO2054 carrying MyoE-GFP and mCherry-SynA, but the density and rapid movement of MyoE-GFP and mCherry-SynA dots made it impossible to determine co-localization unambiguously.

### MyoE localization at the Spitzenkörper is actin dependent but not microtubule dependent

**Figure 5:**
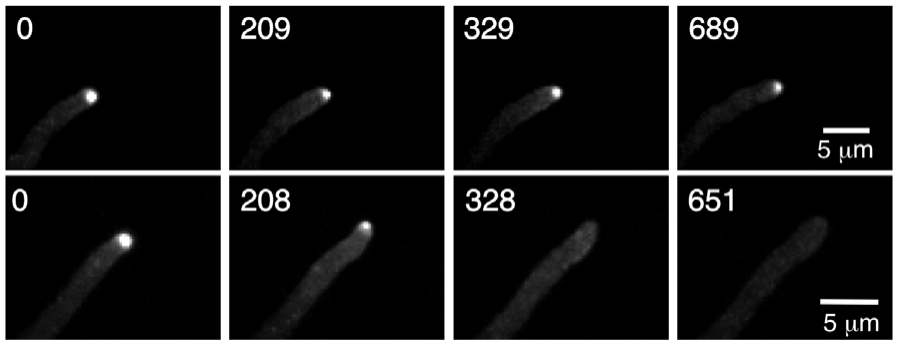
Cytochalasin A causes MyoE-GFP to disperse from the Spitzenkörper. Images are maximum intensity projections of Z-series stacks. Time (in sec) after the addition of DMSO (top row) or an equivalent volume of cytochalasin A dissolved in DMSO to give a final concentration of 1 µg/ml (bottom row). MyoE-GFP continuously localizes to the Spitzenkörper in the solvent control (top row) but disperses in less than 328 sec after the addition of cytochalasin A.

To determine if MyoE localization at the Spitzenkörper is actin dependent, we treated a strain that carries MyoE-GFP with 1 µg/ml cytochalasin A. At this concentration, filamentous actin is depolymerized completely [10]. Cytochalasin A treatment caused rapid disaggregation of the Spitzenkörper MyoE-GFP, indicating that the Spitzenkörper disassembled or that MyoE-GFP dissociated from the Spitzenkörper (Figure 5, Video S4, S5). In fact bidirectional movement was easier to visualize in cytochalasin-treated material than in controls, perhaps because actin-based movement was eliminated. These data indicate that MyoE localization at the Spitzenkörper is actin dependent (or that the structural integrity of the Spitzenkörper is actin dependent), but some of the movement of the MyoE cytoplasmic dots is actin independent and microtubule-based.

**Figure 6:**
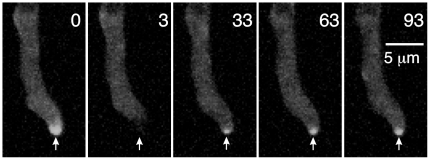
Movement of MyoE-GFP to the Spitzenkörper in the absence of microtubules. Microtubules have been depolymerized with 2.4 µg/ml benomyl time at the upper right in each panel is in seconds. At t = 0, MyoE-GPF is visible at the Spitzenkörper (arrow). Three seconds later after FRAP the MyoE-GFP in the Spitzenkörper is bleached. In spite of the absence of microtubules, MyoE-GFP has moved to the Spitzenkörper 30 sec after FRAP (arrow, t = 33) and it increases in intensity at the Spitzenkörper over the next minute (arrows).

Treatment with 2.4 µg/ml benomyl to depolymerize microtubules did not cause MyoE-GFP to dissociate from the Spitzenkörper (Figure S4). As previously noted [10], however, loss of microtubules caused the tip growth apparatus, including the Spitzenkörper, to be less stably associated with the tip. It often moved to initiate a side branch. The continued presence of MyoE-GFP at the Spitzenkörper could be due to continued transport of MyoE to the Spitzenkörper, or to the MyoE simply remaining at the Sptizenkörper after disassembly of microtubules. To distinguish between these possibilities we examined recovery of MyoE-GFP at the Spitzenkörper after photobleaching. When we kept the size of the bleached region to a minimum, recovery of MyoE-GFP localization to the Spitzenkörper was rapid and the MyoE-GFP signal at the Spitzenkörper was strong (Figure 6). As we increased the size of the bleached region MyoE-GFP signal still recovered at the Spitzenkörper, but the intensity of the signal was weaker, probably because the MyoE-GFP in the vicinity of the Spitzenkörper was also bleached (Figure 6). In the absence of microtubules, we could clearly see movement of MyoE-GFP particles toward the tip (anterograde movement). We did not observe retrograde movement, but because of the density and rapidity of movement of MyoE-GFP particles, we could not rule out the possibility that some retrograde movement occurs. These data in combination reveal that MyoE can move to the Spitzenkörper in the absence of microtubules, and they are consistent with the possibility that microtubules play an important role in moving MyoE to the tip area where it can move on actin filaments to the Spitzenkörper.

### Deletion of *myoE* reduces the hyphal tip extension rate

To determine the functions of MyoE *in vivo*, we examined the effects of the *myoE* deletion by multidimensional microscopy. We first determined tip extension rates by time-lapse imaging of hyphae. A *myoE*Δ strain (LO1935) and parental control (LO1535) were imaged at 25±0.5°C after growth for 24–26 h at 25°C. Z-series stacks were captured at three min intervals. The tip extension rate for the *myoEΔ* strain was 0.19±0.06 µm/min (n = 35), and the rate for the control strain was 0.69±0.13 µm/min (n = 35). The difference in tip extension rates between the two strains was highly significant (P value<0.0001, paired *t*-test).

**Figure 7:**
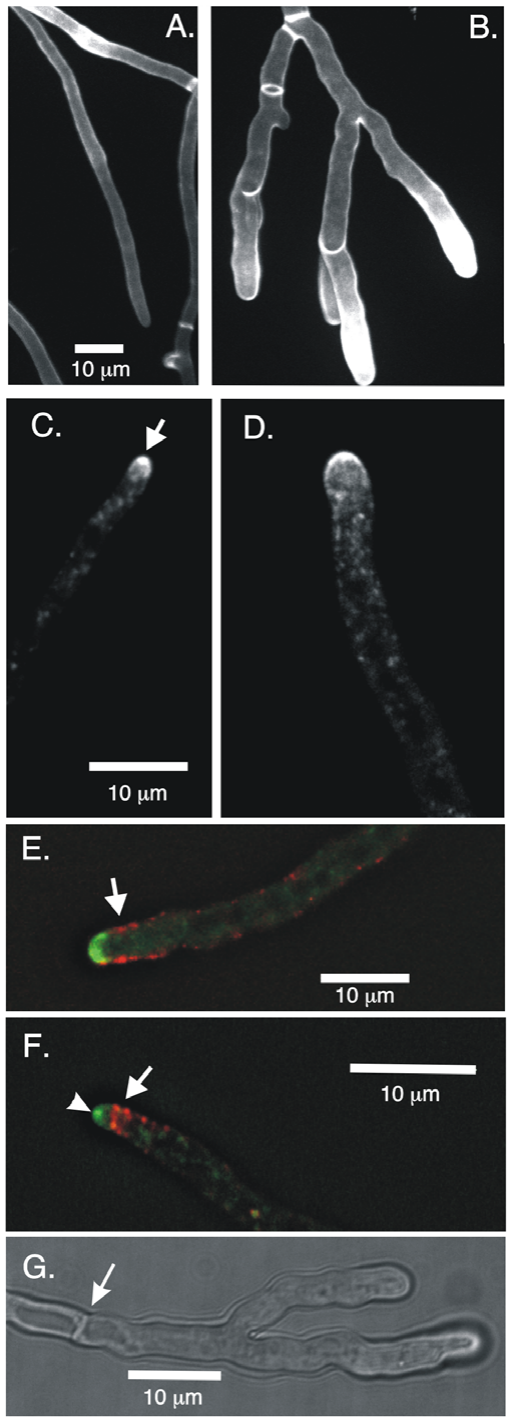
Deletion of myoE alters hyphal morphology and SynA distribution but not the localization of endocytic patches. Panel A shows a *myoE* ^+^ strain and panel B shows a *myoE*Δ strain. Both are stained with 10 µg/ml calcofluor. Hyphae in the *myoE*Δ strain are thicker, vary more in thickness and exhibit more branching near the tip. The amount of chitin staining at the hyphal tip varied from hypha to hypha in wild-type strains as well as *myoB* and *myoE* deletion strains. The difference in staining between A and B is not specific to myoEΔ. Panel C shows GFP-SynA in a *myoE* ^+^ strain. SynA is concentrated into the Spitzenkörper at the hyphal tip (arrow) and is also present at the membrane near the tip. Panel D shows GFP-SynA in a *myoE*Δ strain. SynA is present at the membrane and in puncta in the cytoplasm but is not obviously organized into a Spitzenkörper. Panel E shows the localization of AbpA-mRFP and GFP-SynA in a *myoE*Δ strain. The image is a single focal plane from a deconvolved Z-series stack. AbpA-containing endocytic patches (arrow) localize to the cortex behind the growing tip and in three dimensions form a collar behind the growing tip. Panel F shows a control *myoE* ^+^ strain (LO1548) also expressing GFP-SynA and AbpA-mRFP. The image is a single focal plane from a deconvolved Z-series stack. The ApbA-containing patches (arrow) appear to be organized into a tighter array and the Spitzenkörper is visible (arrowhead). Note that *myoE* ^+^ hyphae are more consistent in diameter along their length than *myoE*Δ hyphae (compare A and B) and that the apices in *myoE*Δ hyphae appear rounder than in *myoE* ^+^ hyphae. A and B are the same magnification as are C and D. Panel G shows branching ahead of the first septum (septum designated with an arrow).

Although the deletion of *myoE* reduced the tip extension rate, it increased hyphal diameter (Figure 7). We measured the diameters of control (LO1535) and *myoE*Δ (LO1935) hyphae growing in liquid culture. To eliminate errors due to variation in diameter along the hypha, for each hypha we measured the diameter 10, 15 and 20 µm behind the hyphal tip and calculated the mean of the three measurements. The diameter of the *myoE* ^+^ strains was 2.60±0.32 µm (n = 50) and the diameter of the *myoE*Δ strain was 4.17±0.44 µm (n = 50). The difference was highly significant (P<0.0001, paired *t*-test).

The actual hyphal growth rate (increase in hyphal volume per unit of time) is a function of both hyphal diameter and hyphal extension rate. To determine if *myoE*Δ inhibited growth we measured both tip extension rates and hyphal diameters for individual *myoE* ^+^ and *myoEΔ* hyphae *and* calculated the increase in volume per unit time for the hyphae. The growth rate for the *myoE* ^+^ hyphae was 3.49±1.17 µm^3^/min whereas the growth rate in the *myoE*Δ strain was 2.59±1.01 µm^3^/min. Although the standard deviations were overlapping, the difference in growth rate was significant (P = 0.0028, paired *t*-test). Deletion of *myoE*, thus, does slightly reduce the hyphal growth rate but, more strikingly, causes hyphae to be thicker and extend more slowly. MyoE, thus, is important for maintaining a thin hypha that extends rapidly, but it is less important for growth itself.

### Hyphal morphology is altered by deletion of *myoE*

As mentioned, the hyphal diameter was greater in *myoE*Δ than in controls. In addition, the hyphal diameter was less consistent along individual hyphae in *myoE* deletants than in *myoE* ^+^ strains (Figure 7). We also noted a significant departure from the wild-type in branching pattern. In the wild-type, branching almost never occurs in the tip cell. A septum must form creating a subapical cell and branches can subsequently extend from subapical cell. We noticed that branching occurred in apical cells in *myoE*Δ strains (Figure 7G) and quantified this by collecting Z-series stacks of many random fields and scoring tip cells for branching before the first septum. In the wild type no branching in the tip cell was seen in 43 tip cells scored. In a *myoE*Δ strain, 22/59 (37%) of hyphal tip cells exhibited branching before the first septum. In many cases the branches seemed to be a simple bifurcation and in a few cases multiple branches extended from the tip cell. In addition, septa appeared to be more numerous and closer together in *myoE*Δ strains than in controls. Finally, although it is difficult to quantify, hyphal tips also appeared to be rounder in *myoE*Δ strains than in controls (Figure 7).

### MyoE is required for SynA localization to the Spitzenkörper but not for SynA movement nor for localization of endocytic patches

**Figure 8:**
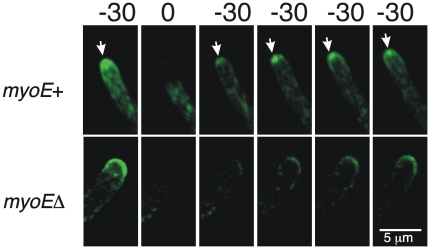
Fluorescence recovery after photobleaching (FRAP) of GFP-SynA in myoE+ and myoEΔ strains. The tips of the two strains were photobleached at T = 0 (sec). In the *myoE*+ strain recovery is rapid. GFP-SynA appears at the tip within 30 sec of photobleaching and quickly localizes to the Spitzenkörper (arrows). This indicates that vesicles with SynA move rapidly to the tip and move through the Spitzenkörper before fusing with the plasma membrane. In the *myoE*Δ strain, the GFP-SynA is also visible at the tip at 30 sec after bleaching. MYOE, thus is not required for movement of GFP-SynA-containing vesicles to the tip. The GFP-SynA does not go through the Spitzenkörper, moreover, but fuses with the plasma membrane in a broad region of the tip.

The morphological alterations caused by *myoE*Δ suggested that tip growth was being altered. As mentioned, SynA is highly concentrated at the Spitzenkörper ([10] and Figure 4A–C, 7C). Since the Spitzenkörper is important for tip growth, we examined GFP-SynA localization in *myoE*Δ strains. We found that although SynA was concentrated near the hyphal apex, it did not localize specifically to the Spitzenkörper in *myoE*Δ strains. It still localized, however, to the apical membrane and to numerous puncta in the cytoplasm (Figure 7D,E, Figure 8, 30 sec time point, Video S6, S7).

The hyphal tip area is very crowded and it is difficult to follow the movement of individual vesicles. To partially circumvent this problem we photo-bleached GFP-SynA vesicles in the hyphal tip region and followed the movement of GFP-SynA from the non-bleached region distal to the tip in *myoE* ^+^ and *myoE*Δ strains. It was still difficult to image individual vesicles, but in the myoE^+^ control, GFP-SynA was visible at the Spitzenkörper 30 sec after the tip was bleached and was visible at the position of the plasma membrane by 60 sec after bleaching (Figure 8). GFP-SynA, and, by inference, exocytic vesicles, thus traveled rapidly from the unbleached subapical area and accumulated specifically at the Spitzenkörper before incorporation into the membrane at the hyphal apex (consistent with the Spitzenkörper functioning as a vesicle supply center). In *myoEΔ* cells, the SynA vesicles also moved rapidly from the unbleached subapical area to the apex (reaching the apex 30 sec after bleaching), but they did not localize to the Spitzenkörper. Rather, they moved directly to the plasma membrane at the tip (Figure 8).

We also examined movement of GFP-SynA in *myoE*Δ and control strains. We observed vigorous and extremely rapid movement of GFP-SynA particles in both strains (Videos S6 and S7). These data allow us to conclude that deletion of *myoE* does not cause a gross reduction of movement of particles containing SynA. Because of the density of particles near the tip and the speed of movement, however, it is difficult to observe SynA particle movement well enough to rule out the possibility that there is partial inhibition of movement or inhibition of movement of a subset of SynA particles.

In *A. nidulans*, an important part of the tip growth apparatus is a ring of endocytic patches just behind the growing tip. This ring apparently is key to recycling components important for tip growth such as SynA and the maintenance of their correct position is actin-dependent [10]. These patches contain, among a number of proteins, AbpA, the *A. nidulans* homolog of Abp1 [10], [30]. To determine if *myoE* is important for the correct positioning of the endocytic patches, we deleted *myoE* in a strain expressing AbpA-mRFP and GFP-SynA. We found that endocytic patches formed a collar behind the growing tip (Figure 7E) as in *myoE* ^+^ cells (Figure 7F). In some cases it appeared that the collar was less well organized than in controls and the AbpA patches were closer to the tip, but this might simply be a consequence of the slower tip extension rates of *myoE*Δ strains.

There is evidence that exocytic vesicles and other SynA containing particles move along both microtubules and microfilaments ([10] and references cited therein) and there is evidence that there is some functional redundancy of the two cytoskeletal systems in *Ustilago maydis* [31]. If microtubule-based movement can compensate for MyoE-powered microfilament-based movement, de-polymerization of microtubules in a *myoE*Δ strain should stop all active particle movement. We consequently determined tip extension rates of *myoE*Δ *and myoE* ^+^ strains after de-polymerization of microtubules by benomyl. The tip extension rate of the *myoEΔ* strain (0.06±0.01 µm/min, n = 16) was significantly slower than that of the *myoE* ^+^ strain (0.22±0.09 µm/min, n = 16) treated identically (p<0.0001, paired *t*-test). Tip extension was, thus, much slower in the absence of both microtubules and MyoE than it was in the absence of either [see [26] for detailed analysis of benomyl effects on tip growth]. This indicates that the two cytoskeletal systems do, indeed, have some functional redundancy. Interestingly, tip growth was not inhibited completely even in the absence of microtubules and *myoE*. It is possible that the residual, extremely slow, growth is simply due to passive diffusion of exocytic vesicles to the plasma membrane and not due to active movement of vesicles by motor molecules along cytoskeletal elements.

## Discussion

There are five genes in *A. nidulans* that encode myosin heavy chains. Three have been studied previously and we now report functional analyses of the remaining two. While our findings are generally consistent with the limited data available from other filamentous fungi [8], [31]–[33], they break new ground with respect to understanding the mechanisms of tip growth and hyphal shape determination in particular, as well as septation.

### MyoE and tip growth

The mechanisms of tip growth and shape determination, and the role of the Spitzenkörper therein, have been the subject of a great deal of research and debate (e.g. [6], [8], [10], [27], [28], [34]–[39]). One important model, the vesicular supply center model, which has been slightly modified over time, contends that the shape of the growing hypha is controlled by the movement of the Spitzenkörper and the fact that vesicles radiate outward from the Spitzenkörper and fuse with the plasma membrane. The frequency of fusion of vesicles, and thus the rate of growth, of any particular portion of the hyphal tip cell would be a function of the distance from the Spitzenkörper. If these premises are correct, the shape of growing hyphae can be predicted by a relatively simple mathematical formula [28], [35]–[37], [39]. While some data support this model, other data (e.g. [10], [40]) seem inconsistent with the model in its most straightforward form.

**Figure 9:**
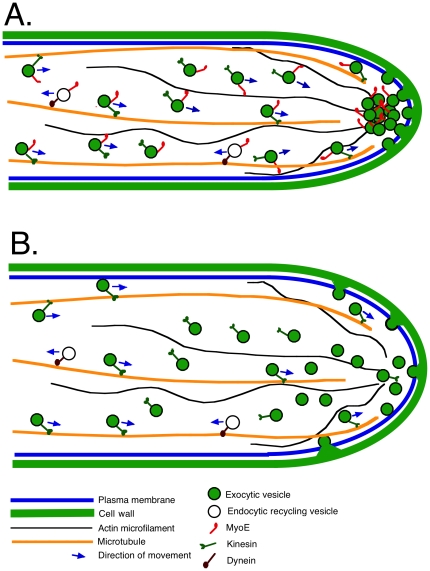
A simplified model for MyoE function at the hyphal tip. A. A *myoE* ^+^ cell. Exocytic vesicles move along microtubules powered by kinesin molecules. (It is likely that several kinesins can carry out this function.) There is a large zone of overlap between microtubules and actin microfilaments. When exocytic vesicles become detached from microtubules, as will generally be the case because of the limited processivity of kinesins, MyoE, on the vesicles will move the vesicles along actin microfilaments, collecting them at the Spitzenkörper. The vesicles then fuse in a fairly small area to the plasma membrane releasing their contents and resulting in hyphal growth. MyoE, vesicle components and, probably, many more proteins are moved in retrograde direction by dynein where they will be reused. B. A *myoE*Δ cell. In the absence of MyoE, exocytic vesicles are not focused into the Spitzenkörper but they are still moved into the hyphal apex area where they fuse with the plasma membrane over a wider area, resulting in hyphae with a greater diameter and lower extension rate. For simplicity, much of the endocytic machinery including endosomes and actin patches has been left out of this model. For a more detailed model of the endocytic machinery please see reference 10.

An illustration of MyoE function at the hyphal tip is shown in Figure 9. Our GFP-SynA photobleaching experiments show that in *myoE* ^+^ strains, exocytic vesicles, as revealed by SynA, traffic from the hypha through the Spitzenkörper into the plasma membrane as predicted by the vesicle supply center model. In the absence of MyoE, however, GFP-SynA, does not accumulate in the Spitzenkörper and the Spitzenkörper, thus, does not act as a vesicle supply center. Tip growth still occurs, however, and the tips have a roughly normal shape. Making the reasonable assumption that SynA is reliable reporter for all classes of exocytic vesicles, it follows that there are mechanisms of growth and shape determination that are independent of vesicles passing through the Spitzenkörper. We have found previously that a tip growth apparatus exists in *A. nidulans* in which several key tip growth components are maintained in a precise spatial relationship [10]. We have now found that key components of the tip growth apparatus, the sites of endocytosis as visualized with AbpA-mRFP and the GFP-SynA at the plasma membrane, maintain approximately normal locations in the absence of MyoE. The tip growth apparatus thus, appears to remain at least partially intact in the absence of MyoE consistent with continued polarized growth.

Tip growth is not normal in *myoE*Δ strains, however. Tip diameter is greater than in controls, the tip diameter is less consistent than in controls, the rate of tip extension is reduced and, the tip appears to be rounder than in controls. These data suggest that while trafficking of vesicles through the Spitzenkörper is not essential for tip growth, the Spitzenkörper does, indeed, act as a vesicle supply center and, as such, plays a major role in determining tip extension rate, hyphal diameter and hyphal shape. Concentrating exocytic vesicles into the Spitzenkörper near the hyphal apex likely results in their fusing into the plasma membrane into a smaller, more defined area, resulting in thinner hyphae of more consistent diameter. It follows that the number of vesicles fusing per unit of time per unit of area at the apex is greater than in the absence of a Spitzenkörper and this results in more rapid tip extension. This, in turn, allows the fungus to explore its environment more rapidly – a considerable selective advantage.

The absence of MyoE causes tip extension to be slower than in controls, but tip diameter is increased and the total growth rate (change in volume per unit time) at the hyphal tip is reduced only about 25% relative to a *myoE*+ control. In addition, since there appear to be more side branches near the tip, the total amount of tip growth may actually be greater in *myoE*Δ strains than in *myoE*+ strains. Since both exocytosis and endocytosis are important for tip growth [6], [7], [10], [28], [35], [36], [41]–[43], and tip growth continues in the absence of MyoE, we can deduce that MyoE is not essential for exocytosis or endocytosis.

Our data also increase our understanding of the mechanisms of movement of vesicles in the tip area. Vesicles can, in principle, move on either of the two cytoskeletal systems, microtubules or actin microfilaments. In understanding movement in the tip area, it is important to remember that in *A. nidulans*, microfilaments extend back from the hyphal apex at least 25 µm (see Fig. 1D in [10]), while microtubules extend from back in the hyphal tip cell to very close to the hyphal apex (reported in many studies e.g. [26], [44]). There is, thus, a considerable zone of overlap of microtubules and microfilaments behind the apex where vesicles can, in principle, move on either of these cytoskeletal elements. When microtubules are depolymerized with benomyl, MyoE continues to move to the Spitzenkörper. The movement of MyoE to the Spitzenkörper, is, thus, not dependent on microtubules and we can deduce that it occurs on microfilaments. Treatment with cytochalasin eliminates localization of MyoE at the Spitzenkörper as predicted if MyoE moves to the Spitzenkörper on microfilaments, but these data are ambiguous because cytochalasin treatment may disrupt the integrity of the Spitzenkörper [10]. The deletion of *myoE* does not eliminate movement of SynA to the hyphal apex but does eliminate its coalescence into the Spitzenkörper. The simplest explanation of these data is that MyoE can power movement of SynA (and, by inference, exocytic vesicles) along actin microfilaments to the Spitzenkörper. MyoE is not limited to moving along microfilaments, however. Although MyoE-GFP puncta do not coalesce to the Spitzenkörper when actin microfilaments are depolymerized with cytochalasin A, they do continue to move to the hyphal apex. This indicates that they can move on microtubules although ample precedence from other systems indicates that MyoE is more much likely to be a passenger on vesicles in this instance than the motor for movement. Since the movement is bi-directional and microtubule motors are unidirectional we can deduce that MyoE is moved by at least two motor molecules. There is very little growth in the absence of both microtubules and MyoE. This indicates that no other myosins or other molecules can substitute for MyoE in actin-based movement of vesicles to the tip and we presume that the extremely slow growth we have observed is due to passive diffusion of vesicles.

In aggregate, our data support and extend the model of Taheri-Talesh et al. [10] and the earlier, similar model of Steinberg [8] as follows. Exocytic vesicles containing SynA and associated with MyoE are transported to the cell tip area on microtubules by kinesins. In the fairly long microtubule/microfilament overlap zone, if exocytic vesicles fall off microtubules, MyoE can move them along actin filaments into the Spitzenkörper before they fuse with the plasma membrane. If microtubules are absent, the supply of vesicles to the tip is reduced, but MyoE can still move vesicles to the Spitzenkörper along actin cables that extend some distance back in the hypha from the apex [10], [30], [45]. As the supply of exocytic vesicles diminishes and is not replenished by movement along microtubules, tip extension slows, as has been demonstrated [10], [26]. In the absence of MyoE, vesicles are still moved along microtubules toward the hyphal apex, but if they fall off of microtubules in the overlap zone, they are not moved into the Spitzenkörper. They can reassociate with microtubules and move to the hyphal apex where they fuse with the plasma membrane over a larger area resulting in a larger tip and consequent hyphal diameter. In the absence of both MyoE and microtubules, there is no active transport of vesicles to the tip and growth is extremely slow. In the absence of microfilaments (i.e. after cytochalsin A treatment) the tip growth apparatus falls apart and growth becomes non-polarized [10], [46]. Related to the cooperation of actin and microtubule cytoskeletons in tip growth, there is evidence in the fungus *U. maydis* that myosin V, and the kin-1 and kin-3 kinesins cooperate in polarized hyphal growth [31] and that the chitin synthase Mcs1 travels along microtubules and actin filaments powered by the kin-1 kinesin and myosin V toward the hyphal tip and moves along microtubules in the opposite direction powered by dynein [47]. In addition, *in vitro*, vertebrate myosin Va (a form of myosin V) can bind to and diffuse along microtubules and that myosin V and kinesin can enhance the processivity of each other [48], [49]. Since we have observed bi-directional movement of MyoE-GFP, we can conclude that a minus-end-directed microtubule motor, such as cytoplasmic dynein, can move MyoE in a retrograde direction and we assume that this allows MyoE to be recycled, to be attached to exocytic vesicles as they are produced well back from the hyphal tip. The transient localization of MyoE to septa suggests it may have a non-essential role in transporting vesicles to the forming septum. Finally, *myoE*Δ causes hyphal branching in tip cells ahead of the first septum and branching often appears to occur by bifurcation. This implies that MyoE plays a role in repressing side branching, although our data give no insights as to mechanism.

In the dimorphic fungus *Candida albicans*, myosin V (which, somewhat confusingly, is designated CaMyo2) is not required for viability but it is required for dimorphic switches and polarized growth [50]. The function of myosin V in *C. albicans* is, thus, not identical to its function in the more typical ascomycete *A. nidulans* although its role in tip growth is probably related to the functions we note for MyoE.

### MyoB and septation

MyoB localizes to forming septa and in its absence, septation is nearly abolished. MyoB, thus, plays a critical role in septation. Since hyphal branching in *A. nidulans* normally occurs through the emergence of new growing tips in subapical cells, we deduce that the lack of branching we noted in *myoB*Δ strains is a consequence of the lack of septation such that subapical cells are extremely rare. Note, however, that some branching occurs even in the absence of septa (Figure 3B). Similarly, the abnormal aggregations of chitin we noted in *myoB*Δ strains may be a consequence of failure of normal deposition of chitin at septa. The reduced branching certainly accounts substantially for the wispy appearance of *myoB*Δ colonies. Note, however, that *myoB*Δ strains (Figure 1) appear to exhibit poor colonial growth beyond what one might expect from simple inhibition of branching. Perhaps there are other deleterious consequences of the lack of septation. Finally, conidiation requires cytokinesis and our data suggest that MyoB is required for cytokinesis during conidiation as well as septation in hyphae.

MyoB is not essential for tip growth. Indeed tip extension is more rapid in *myoB*Δ strains than in controls. This is likely due to the fact that tip extension rates are related to the size and number of nuclei in tip cells (discussion and earlier references in [10]). The absence of septation means that tip cells are extremely large and contain many nuclei, and that there is a large volume of cytoplasm in which components required for tip growth can be synthesized and subsequently transported to the tip. As discussed for MyoE, the fact that tip growth continues robustly in the absence of MyoB, reveals that MyoB is not required for exocytosis or endocytosis.

Although members of the myosin II family have been studied extensively in many organisms, studies in filamentous fungi are extremely limited. The function of myosin II has recently been investigated in the dimorphic fungus *Penicillium marneffei* [33]. The deletion of the myosin II gene in this organism (also designated *myoB*) causes a much less severe growth restriction than the *myoB* deletion in *A. nidulans*. As we have seen in *A. nidulans*, the *P. marneffei myoB* deletion causes abnormal deposition of chitin and causes septation defects, albeit less severe than we have seen in *A. nidulans*. There were some significant differences, however. In *P. marneffei*, the *myoB* deletion causes abnormal branching near the hyphal tip, whereas *myoB*Δ in *A. nidulans* almost completely abolishes branching. In addition, the *myoB* deletion in *P. marneffei* causes abnormal hyphal tip morphology whereas tips in *A. nidulans myoB*Δ strains are morphologically normal. Finally, the deletion of *myoB* causes nuclei to be closer together in *P. marneffei* while this does not appear to be the case in *A. nidulans*. The picture that emerges is that myosin II's in *A. nidulans* and *P. marneffei* have related but not identical functions. The differences may relate, in part, to the dimorphism of *P. marneffei*.

Our data reveal that during septum formation MyoB appears in strings in the area in which septa will form and these strings coalesce to form septal rings. These observations are important for understanding the mechanism of cytokinesis in *A. nidulans* and, by inference, filamentous fungi. Two principal models for contractile ring assembly have been proposed for *S. pombe*, the organism in which, arguably, contractile ring assembly has been studied most completely (reviewed in [51]–[53]). The two models are 1) the aster or spot/leading cable model in which actin filaments are nucleated from a single point at the site of septation and 2) the node, or search, capture, pull and release (SCPR), model in which in components of the contractile ring are pulled together through the action of myosin II moving along actin filaments. The fact that we see strings of MyoB coalescing to form the contractile ring (Figure 2A, Video S1) appears inconsistent with the aster model and provides visible evidence that indicates that the node model, or a variant thereof, operates in *A. nidulans*. In this model, MyoB would interact with actin filaments at the septum and, through its motor activity, pull nodes together to form the contractile ring at the septum and then supply power to constrict the contractile ring. (See Figure 3 in [53] for a drawing of the node model.)

### The functions of myosins in *A. nidulans*

With our data, all myosin heavy chains, or myosin heavy chain-like proteins, have now been functionally analyzed in *A. nidulans*. While new details will undoubtedly emerge, the accumulated data allow a good overview of myosin function. The only essential myosin heavy chain in *A. nidulans* is a type I myosin heavy chain encoded by the *myoA* gene [2]. It is required for secretion, and polarized growth and is important (probably essential) for endocytosis [4], [5]. It appears to be the myosin responsible for exocytosis at the hyphal tip which allows tip extension and for endocytosis behind the hyphal tip that allows recycling of vesicular components. There are two proteins, CsmA and CsmB that have both myosin motor domains and chitin synthase domains [13], [14]. Neither is essential but deletion of either causes reduced growth, osmotic sensitivity and formation of “balloons” (swollen regions) in the hyphae [13], [14]. Deletion or down-regulation of both genes results in complete blockage of growth, but this blockage is partially rescued by 0.6 M KCl [14]. The motor domains are required for function [13], [14], [54]. The available data suggest that the myosin motor domains are involved in targeting the molecules to the hyphal tip and to septa where chitin synthesis is important for strengthening cell walls [13], [14]. MyoB is required for septation and branching and chitin deposition defects may result from the septation defects. MyoE is responsible for moving vesicles into the Spitzenkörper, which allows a more consistent hyphal shape and rapid tip extension.

## Materials and Methods

### Strains and media

All strains used in this work are listed in supplementary material Table S1. The *myoB* deletion was generated by transforming a *myoB::AfpyrG* fusion PCR fragment (as described below) into strain LO1516, and it was analyzed using the heterokayon rescue technique [25]. Solid media were YAG (5 g/l yeast extract, 20 g/l d-glucose, 15 g/l agar, supplemented with 400 µl/l of a trace metal solution [55]) or minimal medium (6 g/l NaNO_3_, 0.52 g/l KCl, 0.52 g/l MgSO_4_⋅7H_2_O, 1.52 g/l KH_2_PO_4_, 10 g/l d-glucose, 400 µl/l trace element solution [55], 15 g/l agar pH 6.0–6.5 and appropriate nutrients to supplement nutritional markers carried by the strains, e.g. 2.5 µg/ml riboflavin, 0.5 µg/ml pyridoxine, and 2.0 µg/ml nicotinic acid). For microscopic observations, conidia were germinated in liquid minimal medium (6 g/l NaNO_3_, 0.52 g/l KCl, 0.52 g/l MgSO_4_⋅7H_2_O, 1.52 g/l KH_2_PO_4_, 10 g/l d-glucose, 400 µl/l trace element solution [55] containing appropriate supplements.

### Gene targeting and deletion

C-terminal tagging was conducted by transforming with linear DNA fragments, which consisted of a selectable marker flanked by two fragments amplified from genomic DNA. The DNA fragments were created by fusion PCR as previously described [56], [57]. To tag *myoB* or *myoE* at the C-terminus with a fluorescent protein sequence such as GFP, a flanking DNA molecule was amplified from the coding sequence of the gene of interest extending about 1000 bp upstream of the stop codon and a similar sized fragment was amplified from the 3′-untranslated region. These fragments were fused to a cassette containing the fluorescent protein sequence and a selectable marker such that the target protein sequence was fused in frame to a linker consisting of five glycine-alanine repeats [58] which was fused, in turn, to the fluorescent protein sequence. The N-terminal tagging of *synA* with GFP or mCherry was carried out as previously reported [10]. To delete the *myoB* or *myoE* gene, flanking DNAs of about 1000 bp were amplified from the 5′- and 3′-untranslated regions of the target genes. The deletion cassettes were generated by fusing the flanks to a selectable marker gene, the *A. fumigatus pyrG* (*AfpyrG*) [59]. Transformation was carried out as described previously [56], [57]. All deletions and fluorescent protein fusions were verified by both diagnostic PCR and Southern hybridizations with the exception of myoB deletants which were verified by diagnostic PCR alone as they grew too poorly to obtain adequate amounts of DNA for Southern hybridizations.

### Diagnostic PCR and Southern hybridizations

Genomic DNA was prepared as described previously [60]. Positive transformants were first verified by diagnostic PCR using outside primers. Subsequently, correct integration of the transforming fragment into the right locus was confirmed by Southern hybridizations using the method of Oakley *et al.* [61] with appropriate radioactively labeled fusion PCR fragments as probes.

### Microscopy and imaging

For live cell imaging, conidiospores were germinated in liquid minimal media in eight-chambered Lab-Tek chambered coverglasses (Nalge Nunc International, Rochester, N.Y.) at 25 or 30°C for 15–20 h. Four systems were used for imaging. Two systems used Olympus IX71 inverted microscopes equipped with mercury illumination sources, Prior shutters, Prior Z-axis drives, and filter wheels. One microscope was equipped with a Hamamatsu ORCA ER camera, and the other with a Hamamatsu ORCA ERAG camera. We used a Semrock GFP/DsRed-2X2M-B dual band “Sedat” filter set [459–481 nm bandpass excitation filter for GFP and a 546–566 nm excitation filter for mCherry, dual reflection band dichroic mirror (457–480 nm and 542–565 nm reflection bands, 500–529 and 584–679 transmission bands) and two separate emission filters (499–529 nm for GFP and 580–654 nm for mCherry imaging)]. Images were acquired with an Olympus 60X 1.42 NA Plan Apochromatic objective using Slidebook Software (Intelligent Imaging Innovations, Denver, CO) or Volocity software (Perkin Elmer) installed on PowerMac computers. For time-lapse two-channel imaging of live cells, Z-series stacks were collected at each time point, and maximum intensity projections from all time points were combined to generate videos using Slidebook or Volocity software. A third imaging system was an Ultraview spinning disk confocal system on a Nikon TE300 inverted microscope with a Hamamatsu ORCA ER camera controlled by Ultraview software (Perkin Elmer-Cetus). The fourth system was a PerkinElmer UltraVIEW VoX spinning disk confocal microscope which was mounted on an Olympus IX71 inverted microscope. It was equipped with a constant-temperature chamber and a piezoelectric stage for rapid Z-axis movement. Two solid-state lasers with wavelengths of 488 and 561 nm were used for excitation. A 60X 1.42 NA objective was used and images were taken with an ORCA ERAG camera. The system was controlled by Volocity software. All systems used for measurements were calibrated with a stage micrometer to ensure accuracy.

### Determination of tip extension rates

With respect to MyoB, a *myoBΔ* deletion strain (it could not be stored permanently and was not assigned a strain number) and a parental control strain (LO1516) were grown in selective liquid minimal media with appropriate supplements at 25°C for 24–28 h. Z-series stacks were captured at three min intervals over 30 min and tip growth extension rates were determined as previously described [26]. Tip extension rates were determined in the same way for *a myoE*Δ strain (LO1935) and *myoE* ^+^ (LO1535) strain except that they were grown for 24–26 h at 25°C before growth rates were determined.

### Inhibitor treatments

Cytochalasin A (Sigma-Aldrich, St. Louis, MO) storage and treatment of hyphae were as previously described [10]. DMSO was used as solvent-only control at the same concentration that was used for cytochalasin A treatments.

To depolymerize microtubules, benomyl was used at a final concentration of 2.4 µg/ml and the treatment was conducted as previously described [26]. Ethanol at the same final concentration that was used for benomyl treatments was used as solvent-only control. In these experiments conidia of strains LO1535 and LO1935 were grown at 30°C for 14–19 h before transferral to 25±1°C. They were allowed to grow at 25°C for at least 10 min before imaging was begun. Data sets were analyzed using the Volocity Quantitation module (PerkinElmer) and Microsoft Excel. The XY coordinates (in pixels) of tip positions of the hyphae were plotted using Volocity software. Fluorescent beads were used as stationary reference points. The XY coordinates of the tip at each time point were exported into Microsoft Excel and the tip growth rates were determined as described previously [26].

### Fluorescence Recovery after Photobleaching (FRAP)

A myoE^+^ strain (LO1535) [10] and a myoEΔ deletion strain (LO1935) expressing GFP-SynA were grown in selective media at 30°C for 15–19 h. They were transferred to 25°C for at least 10 min before the beginning of imaging and photobleaching. Imaging and photobleaching were carried out at 25°C on an Olympus IX71 microscope equipped with a spinning disk confocal system (UltraVIEW VoX; PerkinElmer). The photobleaching was carried out using a UltraVIEW PhotoKinesis Device and a solid-state laser at a wavelength of 488 nm with 70–80% laser power and 8 photobleaching cycles. Images were acquired in 30 sec intervals pre- and post-photobleaching with 10% laser power at a wavelength of 488 nm for 200–300 ms.

## Supporting Information

Table S1 ***Aspergillus nidulans*** **strains used in this study.**
(TIF)

Figure S1 **Domain structure of** ***Aspergillus nidulans*** **myosin heavy chains.**
(TIF)

Figure S2 **Growth of MyoB-GFP fusions at various temperatures.** Three strains are inoculated by center stabs on each plate. At the left is a strain (LO5156) that is wild-type for *myoB*. At the center is a strain (LO1973) that carries GFP fused to MyoB and at the right is a strain (LO2390) that carries the MyoB-GFP fusion as well as mCherry fused to the C-terminus of histone H1. The temperatures and time of growth after inoculation are shown at the right. The growth of the strains carrying MyoB-GFP is indistinguishable from the control at all temperatures and the MyoB-GFP fusion, thus, appears fully functional.
(TIF)

Figure S3 **Growth of strains expressing fusion proteins at various temperatures.** 1: LO5156 (control, parental strain transformed with the *A. nidulans pyrG* gene). 2: LO1975 (MyoE-GFP). 3: LO1540 (mCherry-SynA). 4: LO2054 (MyoE-GFP, mCherry-SynA).
(TIF)

Figure S4 **MyoE localization at the Spitzenkörper remains intact after treatment with 2.4 µg/ml benomyl.** a. MyoE-GFP 5 min before benomyl addition. MyoE-GFP localizes to the Spitzenkörper (arrow). b. 55 min after benomyl addition. MyoE localization to the Spitzenkörper (arrow) remains intact.
(TIF)

Video S1 **Localization of MyoB-GFP during septation.** Nuclei are shown with histone H1-mCherry. As the hypha proceeds toward septation, MyoB begins to accumulate between some nuclei. It is visible as string-like structures. In some cases, MyoB begins to accumulate between two nuclei and then disappears and accumulates between two different nuclei. Eventually the MyoB accumulates into a compact structure extending across the hypha. The structure becomes smaller with time and string-like myoB aggregates can be seen leaving the mass until the structure disappears. Z-series stacks were collected at 30 sec intervals over 40 min. The playback rate is 30 X real time.
(MOV)

Video S2 **A transverse view of MyoB-GFP localization at septum formation.** Z-series stacks were collected over time and rotated using Volocity software to provide a transverse view of septum formation. Particles (presumably cross sections of strings) appear and eventually form a ring. The center of the ring is gradually filled forming a disk. The disk eventually contracts and disappears. Similar images were obtained in instances in which, fortuitously, hyphae grew toward the microscope lens allowing a transverse view of septum formation. Z-series stacks were collected at 30 sec intervals over 26 min. The playback rate is 30 X real time.
(MOV)

Video S3 **Slow motion movement of MyoE-GFP containing structures.** Single focal plane images were captured with a spinning disk confocal microscope at 0.2 sec intervals. Movement of MyoE-GFP structures toward and away from the tip is visible. The playback rate is 0.2 X real time so the movements are slowed 5 X relative to real time.
(MOV)

Video S4 **MyoE-GFP retains its localization at the Spitzenkörper when treated with DMSO (the solvent used for cytochalasin A, see Video S5).** Each image is a projection of a Z-series stack. The stacks were collected at two min intervals except for an interval of 3 min 29 sec when DMSO was added. The video covers a total period of 39 min 29 sec. The playback rate is 120 X real time except for the interval when DMSO was added. The hypha moved rapidly to the right when the DMSO was added and the specimen continued to drift slightly to the left after the addition, but the Spitzenkörper localization of MyoE-GFP was not altered.
(MOV)

Video S5 **MyoE-GFP disperses from the Spitzenkörper after cytochalasin A treatment.** Each image is a projection of a Z-series stack. The stacks were collected at two minutes intervals except for an interval of 3 min 28 sec when cytochalasin was added and 1 min 23 sec between frames 8 and 9. The video covers a total period of a period of 20 min 51 sec. Cytochalasin was added after time point 6 and the MyoE-GFP dispersed rapidly from the Spitzenkörper.
(MOV)

Video S6 **GFP-SynA localizes to the Spitzenkörper in a myoE^+^ strain. Note also movement of particles along the hypha.** Single focal plane images were collected at 0.112 sec intervals. Playback is 0.112 X real time so movement is slowed by almost 9 X relative to real time.
(MOV)

Video S7 **GFP-SynA does not localize to the Spitzenkörper in a myoEΔ strain.** GFP-SynA localizes to the membrane at the tip and to particles in the cytoplasm that still move vigorously but it does not localize to the Spitzenkörper. Single focal plane images were collected at 0.112 sec intervals. Playback is 0.112 X real time so movement is slowed by almost 9 X relative to real time.
(MOV)
