# Supplementary figures and images for: The Functions of Myosin II and Myosin V Homologs in Tip Growth and Septation in Aspergillus nidulans
Source: PLoS One. 2012 Feb 16;7(2):e31218. doi: 10.1371/journal.pone.0031218 (PMC3281053; doi:10.1371/journal.pone.0031218)

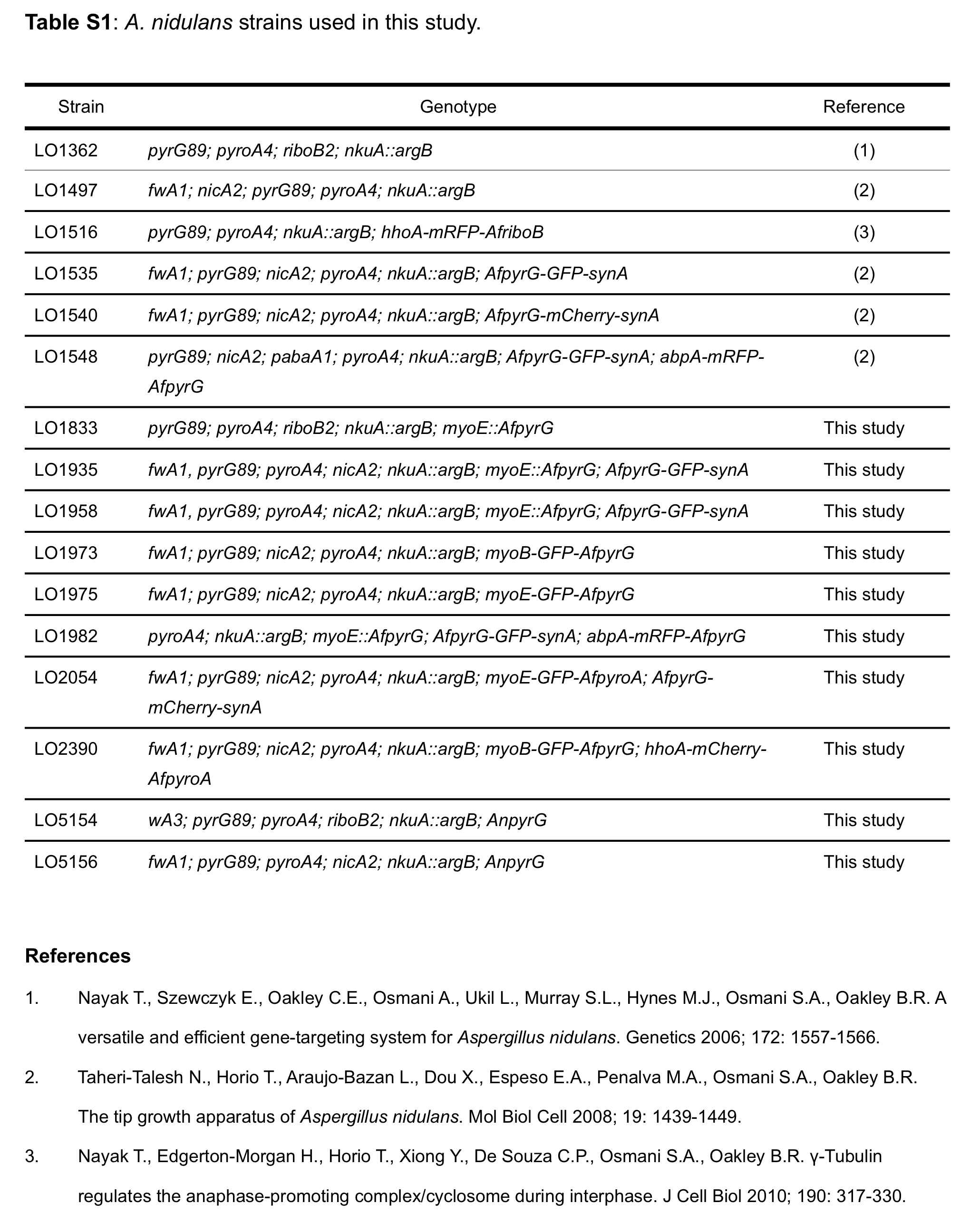

Supplement: Table S1 — Aspergillus nidulans strains used in this study. (TIF) [file pone.0031218.s001.tif]

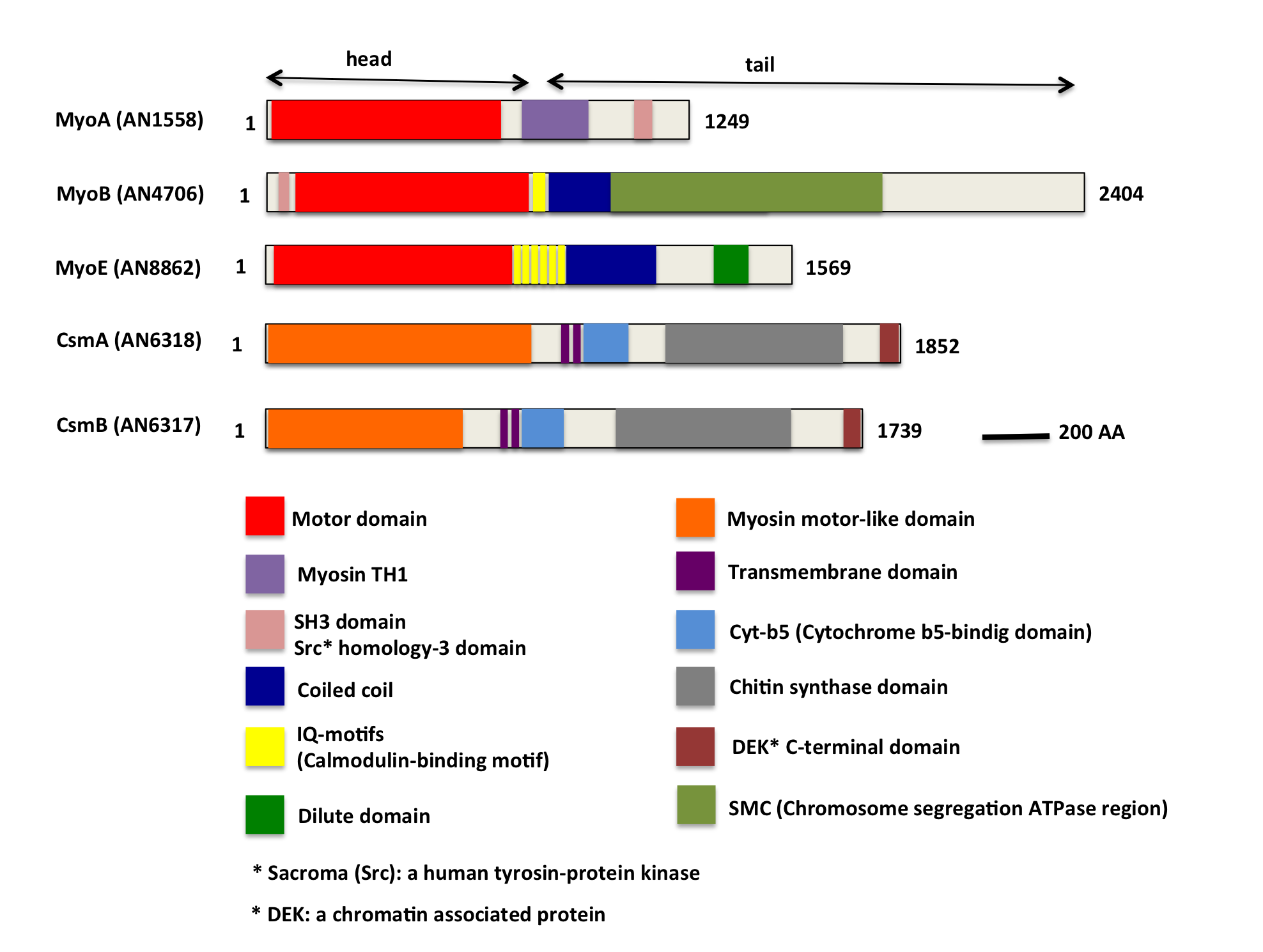

Supplement: Figure S1 — Domain structure of Aspergillus nidulans myosin heavy chains. (TIF) [file pone.0031218.s002.tif]

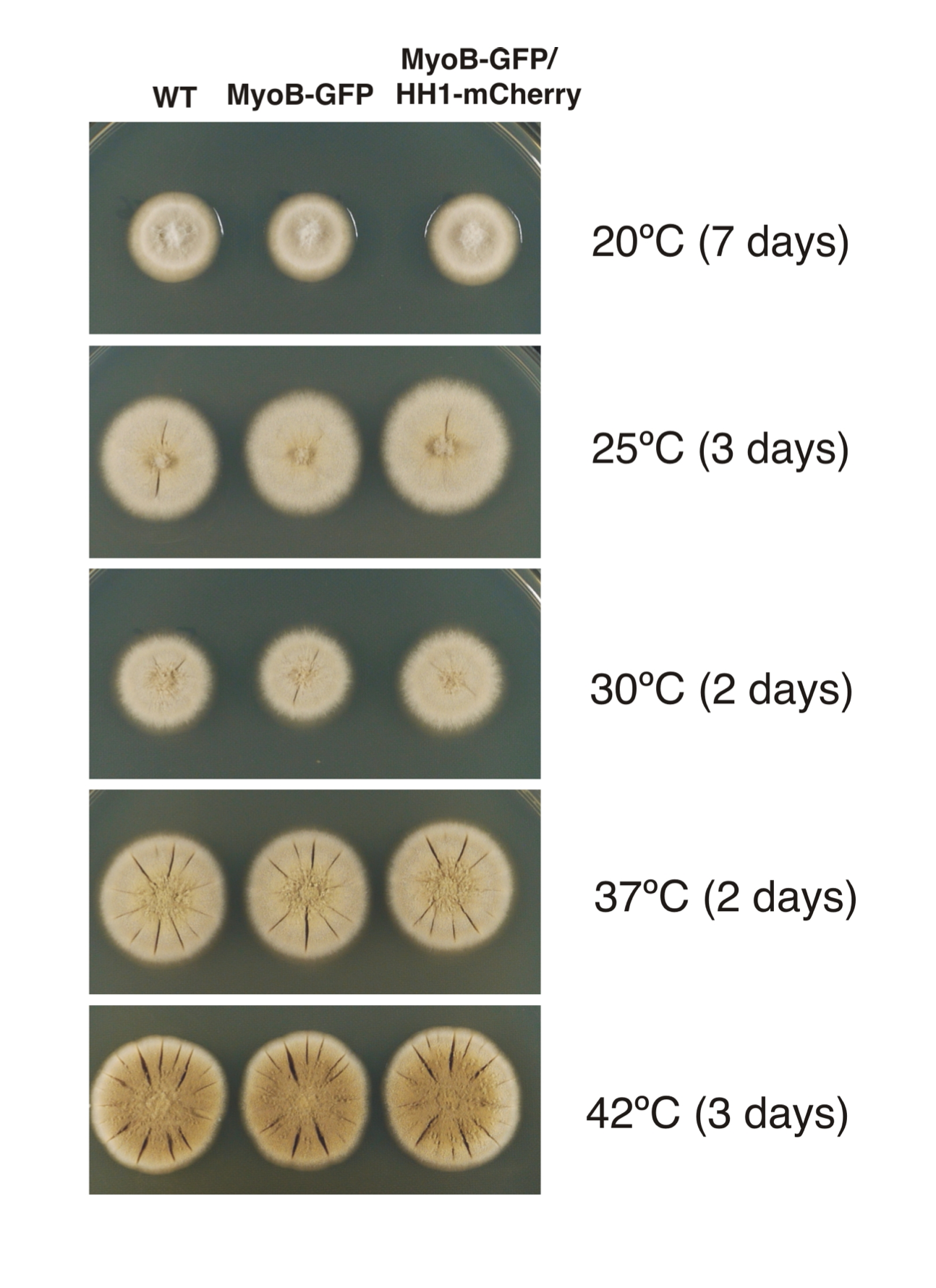

Supplement: Figure S2 — Growth of MyoB-GFP fusions at various temperatures. Three strains are inoculated by center stabs on each plate. At the left is a strain (LO5156) that is wild-type for myoB. At the center is a strain (LO1973) that carries GFP fused to MyoB and at the right is a strain (LO2390) that carries the MyoB-GFP fusion as well as mCherry fused to the C-terminus of histone H1. The temperatures and time of growth after inoculation are shown at the right. The growth of the strains carrying MyoB-GFP is indistinguishable from the control at all temperatures and the MyoB-GFP fusion, thus, appears fully functional. (TIF) [file pone.0031218.s003.tif]

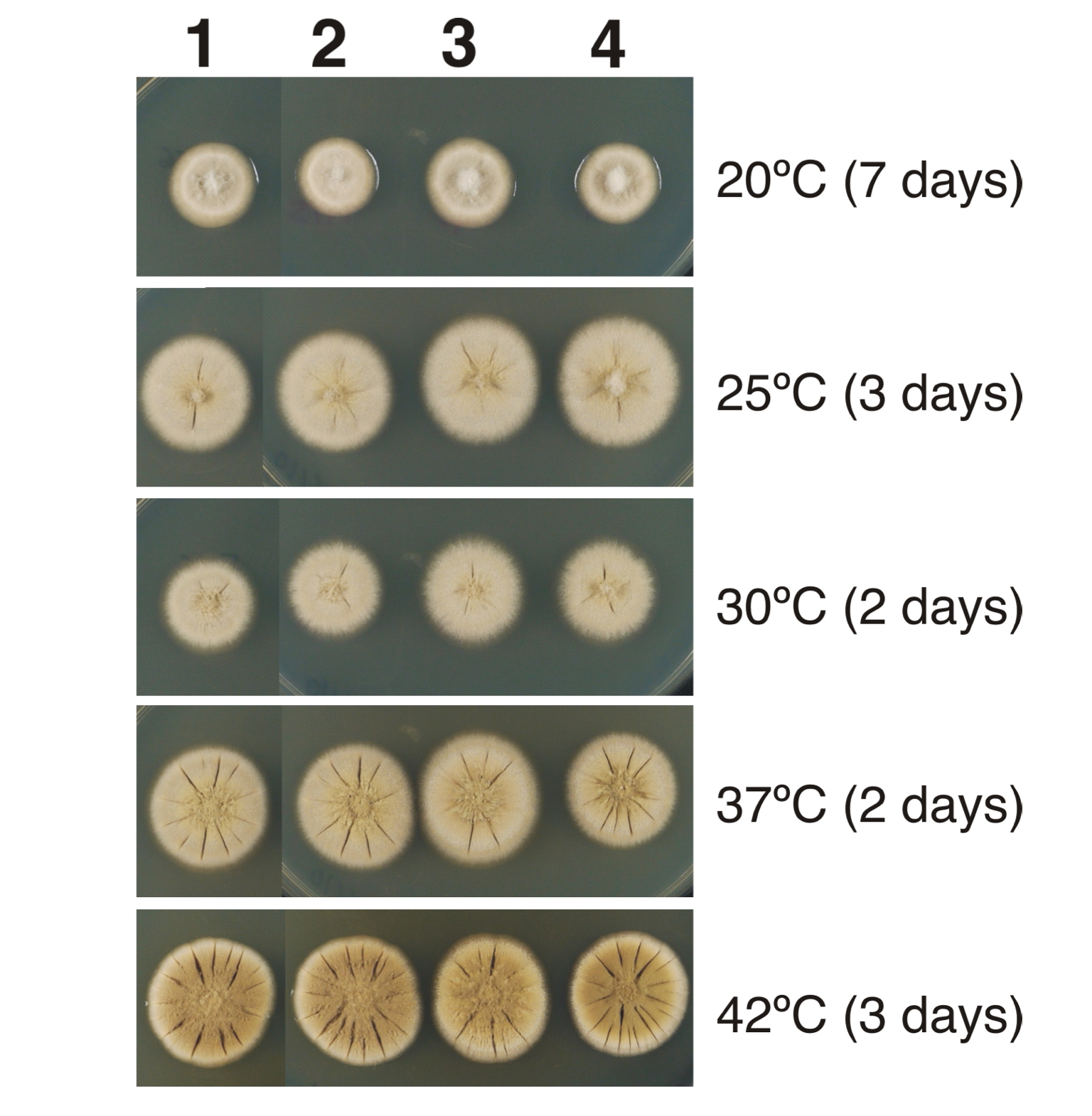

Supplement: Figure S3 — Growth of strains expressing fusion proteins at various temperatures. 1: LO5156 (control, parental strain transformed with the A. nidulans pyrG gene). 2: LO1975 (MyoE-GFP). 3: LO1540 (mCherry-SynA). 4: LO2054 (MyoE-GFP, mCherry-SynA). (TIF) [file pone.0031218.s004.tif]

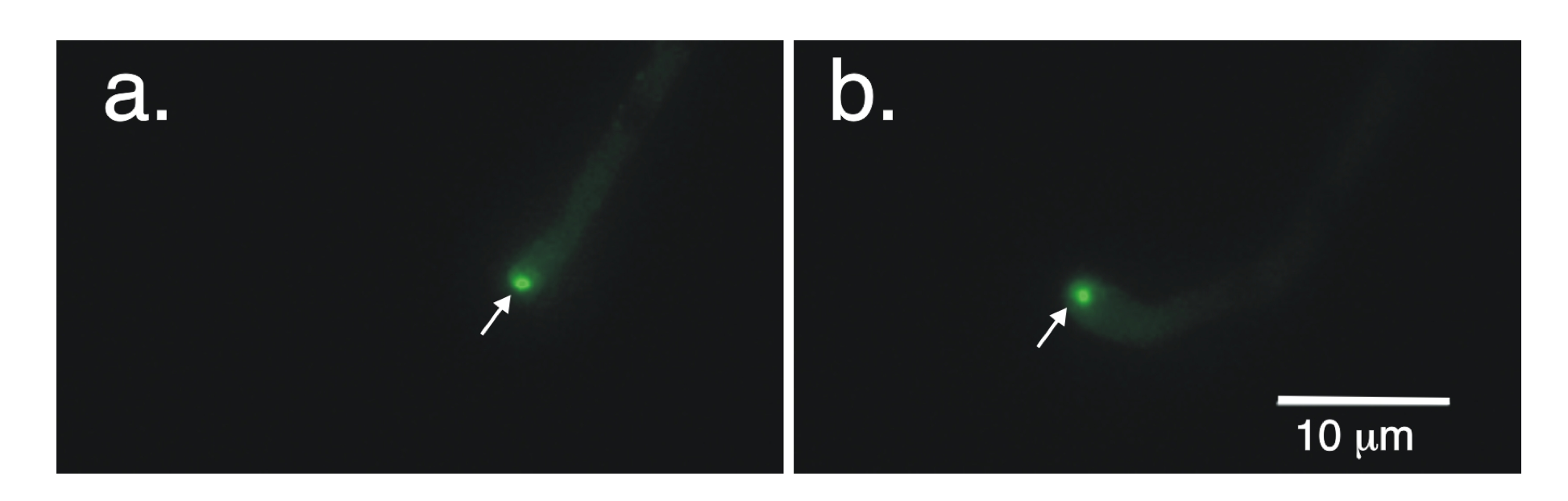

Supplement: Figure S4 — MyoE localization at the Spitzenkörper remains intact after treatment with 2.4 µg/ml benomyl. a. MyoE-GFP 5 min before benomyl addition. MyoE-GFP localizes to the Spitzenkörper (arrow). b. 55 min after benomyl addition. MyoE localization to the Spitzenkörper (arrow) remains intact. (TIF) [file pone.0031218.s005.tif]
